# Time-series biological responses toward decellularized bovine tendon graft and autograft for 52 consecutive weeks after rat anterior cruciate ligament reconstruction

**DOI:** 10.1038/s41598-022-10713-y

**Published:** 2022-04-25

**Authors:** Masafumi Itoh, Hiroki Imasu, Kazuya Takano, Mitsuo Umezu, Ken Okazaki, Kiyotaka Iwasaki

**Affiliations:** 1 Cooperative Major in Advanced Biomedical Sciences, Joint Graduate School of Tokyo Women’s Medical University and Waseda University, 2-2 Wakamatsucho, Shinjuku, Tokyo 162-8480 Japan; 2 Department of Orthopaedic Surgery, Tokyo Women’s Medical University, 8–1, Kawada-Cho, Shinjuku-ku, Tokyo 162-8666 Japan; 3 Department of Modern Mechanical Engineering, Waseda University, 3-4-1 Ohkubo, Shinjuku, Tokyo 169-8555 Japan; 4 Department of Integrative Bioscience and Biomedical Engineering, Graduate School of Advanced Science and Engineering, Waseda University, 2-2 Wakamatsucho, Shinjuku, Tokyo 162-8480 Japan

**Keywords:** Biomaterials, Tissue engineering

## Abstract

There is an essential demand for developing biocompatible grafts for knee anterior cruciate ligament reconstruction (ACLR). This study investigated cell infiltration into decellularized bovine tendon xenografts using a rat knee ACLR model. Twelve-week-old Sprague–Dawley rats were used. At weeks 1, 2, 4, 8, 16, 26, and 52 (each period, n = 6) after ACLR, rats receiving decellularized bovine tendon (group D, n = 42) or autologous tendon (group A, n = 42) as grafts underwent peritibial bone tunnel bone mineral density (BMD), histological, and immunohistological assessments. BMD increased over time in both the groups until week 16 and then remained unchanged without exhibiting significant differences between the groups. Initially, cellularity in group D was lower than that in group A; however, by weeks 4–8, both the groups were comparable to the native anterior cruciate ligament group and cellularity remained unchanged until week 52. Initially, group A had more M1 macrophages, indicating inflammation, whereas group D had more M2 macrophages, indicating tissue regeneration. Nonetheless, the M1 and M2 macrophage counts of both the groups were comparable at most times. This study revealed the excellent recellularization and tendon–bone integration abilities of decellularized tendons using a cross-species model.

## Introduction

Anterior cruciate ligament (ACL) injury is one of the most common and troublesome injuries of the knee^1^. Due to its poor self-healing ability, majority of patients who want to return to sports must undergo ACL reconstruction (ACLR) to restore knee stability^2^. In the United States, 200,000 ACL injuries are expected to occur annually, and the direct cost of ACLR is approximately $3 billion per year^3^. For ACLR, three types of grafts are used—autografts^4^ (using the patient’s tissue), allografts^5^ (using cadaveric tissue), and artificial ligaments^6^. Autograft is the gold standard graft for ACLR because the clinical outcomes of using artificial ligaments are relatively poor^7^ and allografts present specific drawbacks such as disease transmission^8^ and tissue damage caused by the use of gamma irradiation for sterilization^9^. Furthermore, there are still unresolved issues with autografts as well. First, donor site morbidity, such as anterior knee pain and patellar fracture caused by harvesting bone–patellar tendon–bone grafts^10,11^, and skin sensory changes in the lower leg caused by harvesting medial hamstring tendons^12^ have been reported. Second, the graft’s volume is insufficient. This issue may arise not only in multiple ligament reconstruction or revision ACLR but also in primary ACLR. ACLR using hamstring grafts < 8 mm in diameter has a higher failure rate^13,14^. Furthermore, if the patient is short in stature, the volume of the hamstring may be lesser^15^, resulting in a graft < 8 mm in diameter^16^. Third, harvesting autografts from the patient’s body would be a time-consuming and labor-intensive process if performed during surgery.

The use of decellularized tissues obtained from animal sources is attractive because they have a biological tissue matrix unique to target tissues. The extracellular matrix (ECM) of the native tendon tissue consists of type 1 collagen fibril bundles with typical alignment and anisotropic orientation^17^; both these features support tendon differentiation in progenitor cells^18,19^. Furthermore, many ECMs are conserved in various species^20^. Thus, the present study compared the time-series biological responses of a rat ACLR model to decellularized bovine tendon xenografts and autologous flexor digitorum longus tendon grafts for up to 52 weeks as well as their corresponding tendon–bone integration abilities.

## Results

### Gross appearance of the femur-graft–tibia complex at each time point

**Figure 1:**
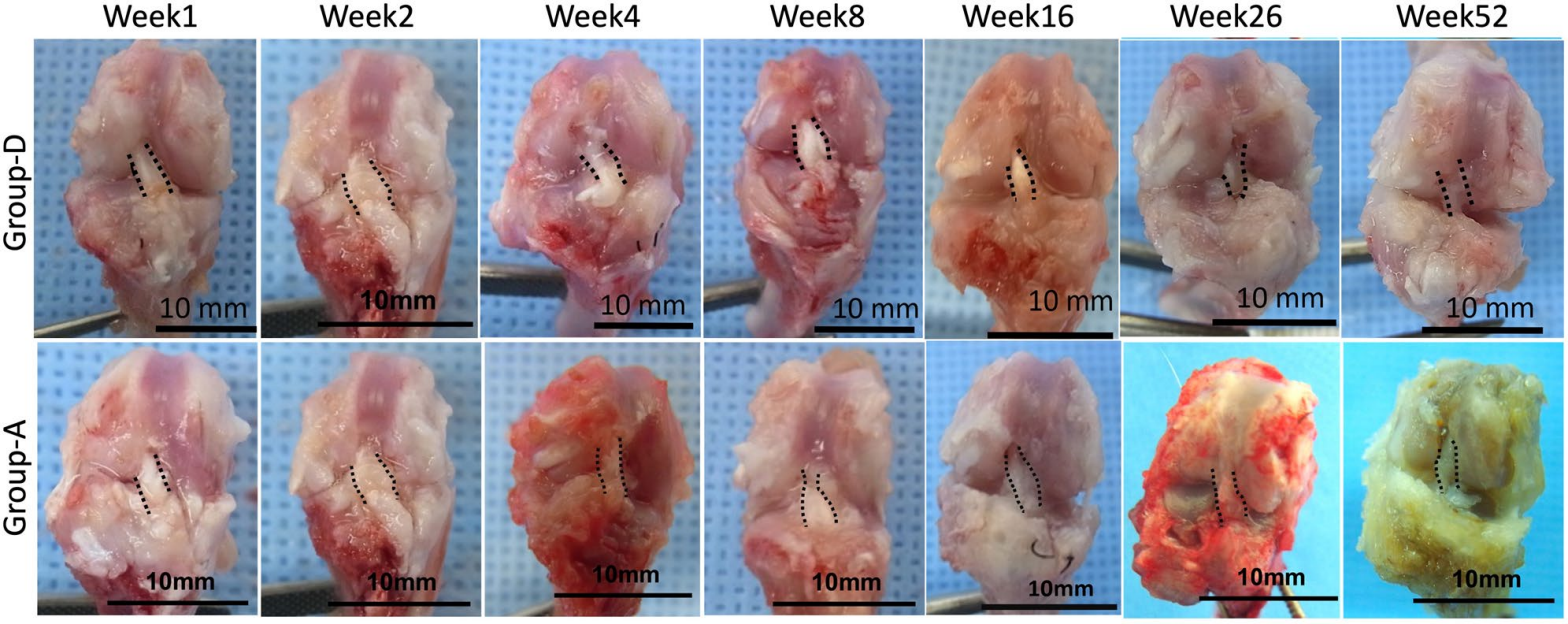
The gross appearance of the right knee at each time point in representative cases from groups D and A. The black dashed line shows the outline of the graft. *Group D* decellularized bovine tendon graft, *group A* autologous tendon graft.

Figure 1 depicts the gross appearance of the right knee at each time point for representative cases from groups D and A. The decellularized xenograft tissues were retained for 52 weeks in the knee joint in a manner similar to that of the autografts.

### BMD of the peritibial tunnel

**Figure 2:**
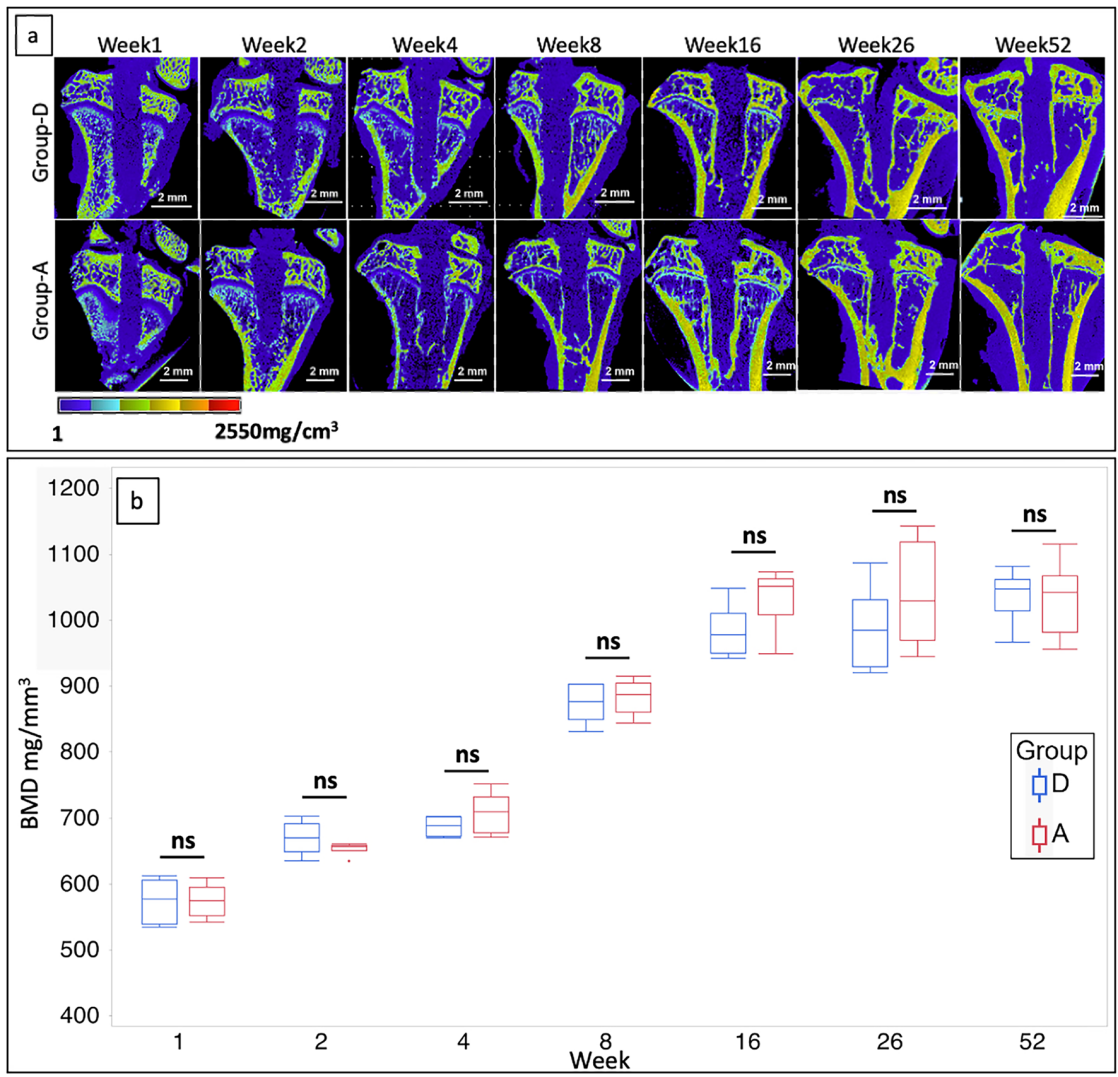
Time-series analysis of BMD at the interface between graft and peritibial bone tunnel. (**a**) Micro-CT images of slices parallel to the tibial tunnel at each time point in each group. (**b**) Box plots showing the BMD of the peritibial tunnel for each group at each time point. (n = 6, for each period in each group). *BMD* bone mineral density, *CT* computed tomography, *group D* decellularized bovine tendon graft, *group A* autologous tendon graft, *ns* not statistically significant.

Microcomputed tomography (micro-CT) images of the slices parallel to the tibial tunnel obtained at each time point for each group are shown in Fig. 2a. There was no significant enlargement of the tibial tunnel in either of the groups over time. In each group, the BMD of the peritibial tunnel increased over time for up to week 8 and plateaued after week 16 (Fig. 2b). There was no significant difference between the groups at each time point. The BMD of group D at weeks 4 (Supplementary Video S1) and 52 (Supplementary Video S2) revealed that there was no enlargement in tunnel diameter at week 52, and BMD at the graft–bone interface increased at week 52 compared with week 4.

### Cellularity in the intra-articular graft and intratibial tunnel graft

**Figure 3:**
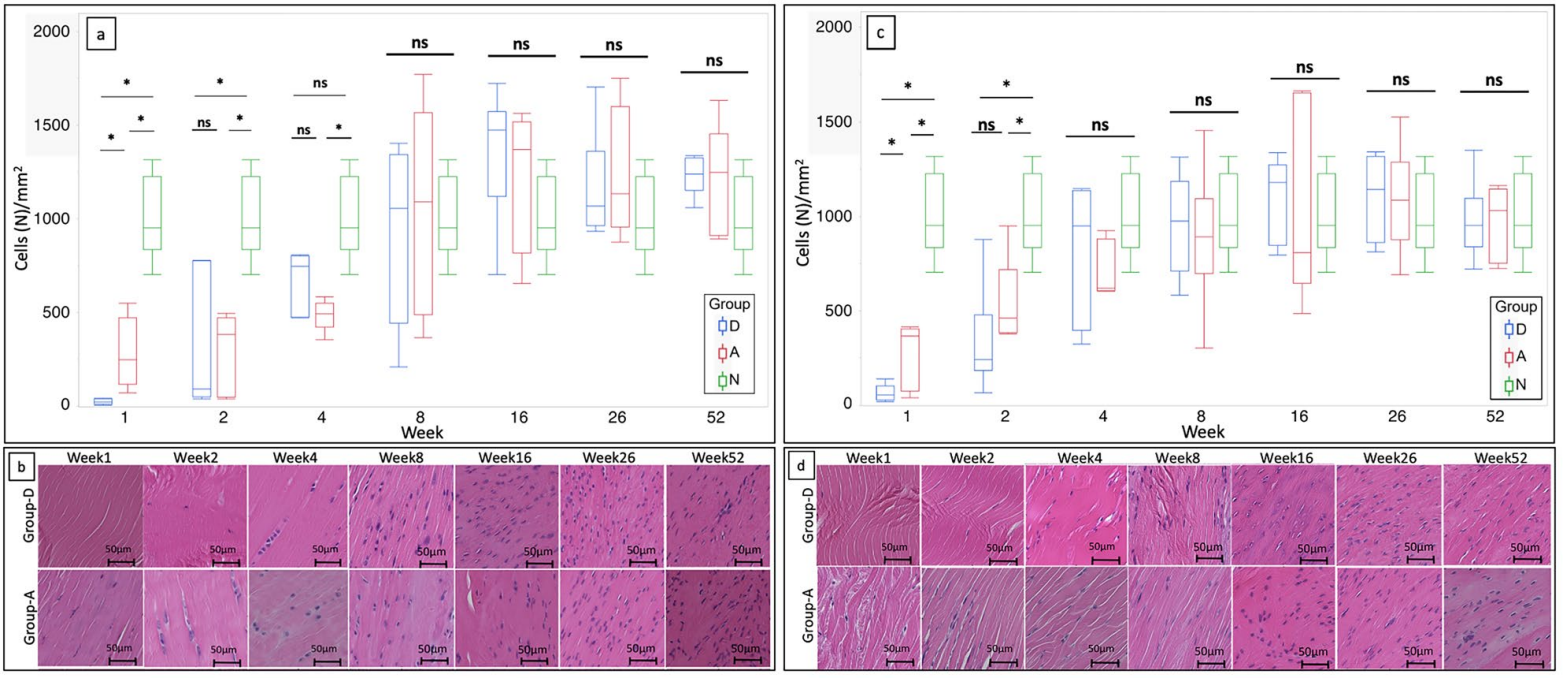
Comparison of the cellularity of the decellularized bovine tendon and autologous tendon in rat ACL reconstruction for up to 52 weeks of implantation. (**a**) Box plots depicting the count of cells in the intra-articular grafts of groups D and A at each time point. The number of cells in native ACL are shown as group N (n = 6, for each period in each group). (**b**) Representative hematoxylin–eosin staining showing cell infiltration in the intra-articular graft at various time points in groups D and A. (**c**) Box plots showing the count of cells in the intratibial tunnel grafts of groups D and A at each period. The number of cells in native ACL are shown as group N. (n = 6, for each period in each group). (**d**) Representative hematoxylin–eosin staining showing cell infiltration in the intratibial tunnel graft at various time points in groups D and A. *ACL* anterior cruciate ligament, *group D* decellularized bovine tendon graft, *group A* autologous tendon graft, *group N* native ACL, *statistically significant (p < 0.05), *ns* not statistically significant.

The graft between the apertures of the femoral and tibial tunnels was defined as the “intra-articular graft”. The term “intratibial tunnel graft” refers to a graft that enters the tibial tunnel deeper than the tibial tunnel aperture. Figure 3a presents the box plots showing the count of cells in the intra-articular graft of groups D and A at each period and group N (native ACL, day 0). At week 1, the cellularity of group D was lower than that of group A (p = 0.01), and the cellularity of both the groups was lower than that of group N (p = 0.007, p = 0.01, respectively). At week 2, there was no longer a significant difference between groups A and D due to increased cellularity in group D. Nonetheless, the cellularity of both groups D and A was lower than that of group N (p = 0.007 and p = 0.02, respectively). At week 4, there was no significant difference between groups D and N due to increased cellularity in group D. On the other hand, the cellularity was significantly lower in group A than in group N (p = 0.007). At week 8, because the cellularity of both groups A and D increased further, no significant difference was observed between the three groups. After week 16, the cellularity of the three groups was comparable and remained unchanged thereafter. Figure 3b depicts cell infiltration into the intra-articular graft at various time points in representative cases from groups D and A. At week 1, a small number of infiltrated cells were observed in group D. At week 2, cell infiltration increased in group D, which further increased at week 4. Cell infiltration increased further in both the groups by week 8. Furthermore, cells with oval or rod-shaped nuclei appeared in the graft at week 8 in both the groups. Subsequently, the proportion of cells in the graft with oval or rod-shaped nuclei increased even more, and they were aligned longitudinally in parallel with regularly oriented fibers.

Figure 3c presents the box plots showing the count of cells in the intratibial tunnel graft of groups D, A, and N. At week 1, the count of cells in group D was lower than that in group A (p = 0.04), and the cell count in both groups A and D was lower than that in group N (p < 0.0001, p < 0.0001, respectively). At week 2, the cell count of both groups D and A increased, and no significant difference was found between the two groups. The number of cells in both groups D and A, however, was significantly lower than that in group N (p = 0.01, p = 0.0008, respectively). After week 4, there was no significant difference between the three groups, and this was maintained until week 52. Figure 3d depicts cell infiltration into the intratibial tunnel graft at different time points in representative cases of groups D and A. At week 1, there was a small number of infiltrated cells in group D. At week 2, cell infiltration increased in both the groups. At week 4, cell infiltration further increased in both the groups, and their cellularities appeared to be comparable. At week 8, cells with oval or rod-shaped nuclei appeared, which were then aligned longitudinally and oriented in parallel with collagen fibers in both the groups. After week 26, almost all cells had rod-shaped or oval nuclei that aligned longitudinally in parallel with regularly oriented fibers in groups D and A. No avascular fibrous capsules were formed in the grafts of these groups, regardless of the time.

### Changes in the graft–tibial tunnel interface over time

**Figure 4:**
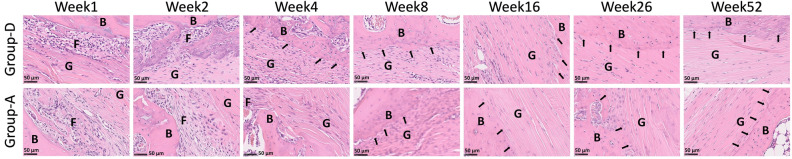
Representative hematoxylin–eosin staining images of the graft–tibial tunnel interface in groups D and A taken at various time points; the black arrow indicates a Sharpey-like fiber. *B* bone, *T* tendon, *F* fibrous tissue, *group D* decellularized bovine tendon graft, *group A* autologous tendon graft.

Figure 4 depicts representative hematoxylin–eosin staining showing the graft–tibial tunnel interface of groups D and A at various time points. At week 1, group D showed a smaller width of loose fibrous tissue at the graft–tibial tunnel interface compared with group A. At week 2, this width decreased in group A and was further reduced in group D. At week 4, the fibrous tissue at the graft–tibial tunnel interface almost disappeared in group D, whereas a small amount of it remained in group A. Moreover, Sharpey-like fibers connecting the graft to the bone appeared in group D but not in group A. At week 8, the amount of such fibers increased in group D, and they also appeared in group A, where their amount increased at week 16. After 16 weeks, the graft–tibial bone tunnel interface fused, and the interface was maintained in both the groups.

### Count of M1 and M2 macrophages in the intra-articular and intratibial tunnel grafts

**Figure 5:**
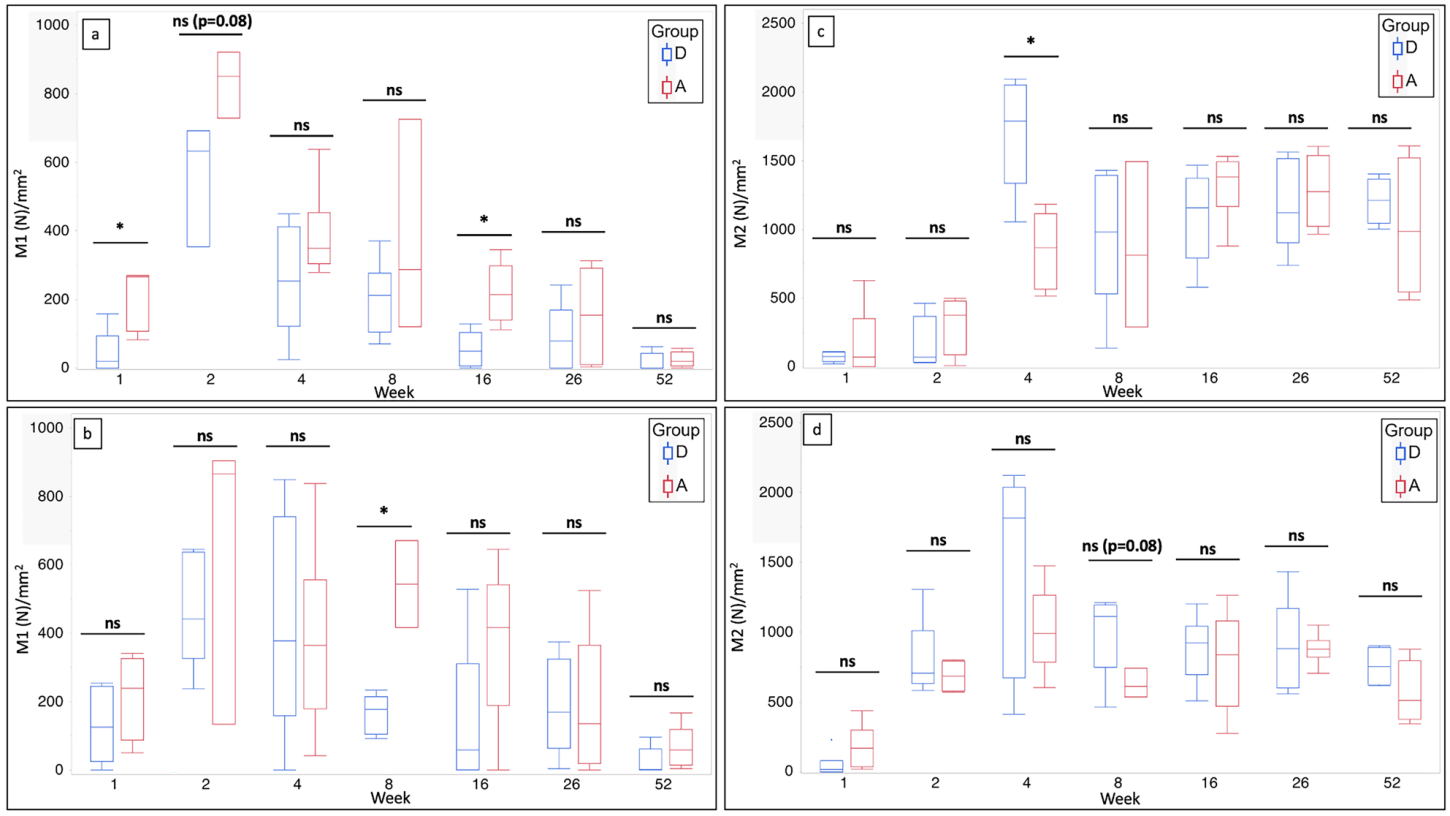
Comparison of the count of M1 and M2 macrophages that appeared in the decellularized and autologous tendons in rat anterior cruciate ligament reconstruction within up to 52 weeks after implantation. (**a**) Box plots showing the count of M1 macrophages in the intra-articular grafts of groups D and A at each time point (n = 6, for each period in each group). (**b**) Box plots showing the count of M1 macrophages in the intratibial tunnel grafts of groups D and A at each time point (n = 6, for each period in each group). (**c**) Box plots showing the count of M2 macrophages in the intra-articular grafts of groups D and A at each time point (n = 6, for each period in each group). (**d**) Box plots showing the count of M2 macrophages in the intratibial tunnel grafts of groups D and A at each time point (n = 6, for each period in each group). *Group D* decellularized bovine tendon graft, *group A* autologous tendon graft, *group N* native anterior cruciate ligament; *statistically significant (p < 0.05), *ns* not statistically significant.

The M1 macrophage counts in the intra-articular grafts of groups D and A at each time point are shown in Fig. 5a. The number of M1 macrophages in group D was significantly lower than that in group A at week 1 (p = 0.03). The M1 macrophage count was significantly higher at week 2 than at week 1 in both the groups (p = 0.04 and 0.04, respectively). It tended to be higher in group A than in group D; however, the difference was not significant (p = 0.08). At weeks 4 and 8, no significant difference was present between the groups in terms of M1 macrophage counts. The M1 macrophage count in group D was significantly lower than that in group A at week 16 (p = 0.002). At weeks 26 and 52, no significant difference was observed between the groups in terms of the M1 macrophage count. After the mid-term, a nonsignificant downward trend in the count of M1 macrophages was observed in both the groups.

The count of M1 macrophages in the intratibial tunnel graft of groups A and D at each time point is shown in Fig. 5b. At week 1, no significant difference was found between groups D and A. In comparison to week 1, the M1 macrophage count in group A increased numerically but with no statistical significance in week 2, whereas group D showed a significant increase (p = 0.003). At weeks 2 and 4, there was no significant difference between the groups. The M1 macrophage count in group D was significantly lower than that in group A at week 8 (p = 0.046). After week 16, there was no significant difference between the groups. The M1 macrophage count in group A decreased numerically with no statistical significance at week 52 compared to week 26, whereas that of group D decreased with statistical significance (p = 0.02).

The M2 macrophage count in the intra-articular graft of both the groups at each time point is shown in Fig. 5c. At weeks 1 and 2, there was no significant difference between the groups. At week 4, the M2 macrophage count increased significantly in both the groups compared to that at week 2 (p = 0.01, p = 0.01, respectively). Furthermore, the M2 macrophage count in group D was significantly higher than that in group A (p = 0.002). After week 8, there was no significant difference between the groups.

The M2 macrophage count at each time point in the intratibial tunnel graft of both the groups is shown in Fig. 5d. At week 1, there was no significant difference between the groups. At week 2, the M2 macrophage count in both the groups increased significantly compared to that at week 1 (p = 0.01, 0.007, respectively), with no significant difference between the groups. At week 8, it tended to be higher in group D than in group A, with no statistical significance (p = 0.08). After week 8, the M2 macrophage count remained unchanged and was comparable between the groups.

## Discussion

The most important finding of this study was that decellularized bovine tendon xenografts were repopulated with recipient rat cells and the grafts were retained for 52 weeks, as were autologous grafts in the rat ACLR model. The decellularized tendons maintaining the native tissue structure without any cellular component induced an M2-dominant host response and earlier cell infiltration into the xenografts compared with the autografts. In contrast, the autografts with cellular components resulted in M1-type inflammation and cell infiltration was slower in group A than in group D.

The cellularity of the graft of the intra-articular and intratibial regions became comparable in both the groups after weeks 8 and 4, respectively, and equivalent to that of native ACL. It is important to note that the cellularity of group D did not exceed that of group A at weeks 1 and 2. If the decellularization was inadequate, there may be many inflammation-derived cells infiltrating the xenograft in the early stage, and the cellularity of the decellularized xenograft might exceed that of the autologous graft^21–23^. The fact that cellularity in both the intra-articular and intratibial tunnel grafts was lower in group A at weeks 1 and 2 than in group N could be attributed to graft necrosis^24–28^. Kondo et al.^28^ reported that 2 weeks after ACLR in sheep, the core of an autologous semitendinosus graft was nearly acellular. They concluded that graft necrosis occurred at week 2, followed by revascularization and cellular infiltration over time, resulting in graft regeneration. The cellularity in group A was lowest at week 1 but not acellular. In terms of tissue regeneration, rats are most likely ahead of sheep^29^. Hence, autologous tendon cellularity may be at its lowest before week 1 or between weeks 1 and 2. Furthermore, in sheep models^28^, the orientation of cells having rod-shaped or oval nuclei began to appear in the grafts at week 12, whereas in the current study, the orientation of cells began to appear at week 8. It is also important to note that the cellularity in group D was maintained after reaching a level equivalent to that of group N. If the number of cells in group D increased and far exceeded that in group N, the decellularized graft might become cancerous^30–32^. Therefore, decellularized bovine tendon grafts were considered safely integrated as a new ACL in rats.

The BMD of the peritibial tunnel of groups D and A was comparable at each time point; it increased up to week 16 and then stabilized. One of the causes of early graft failure is the failure of osseointegration into the graft^33^. The success of the healing phase of osseointegration into the graft coincides with an improvement in load to failure^24^. Therefore, tendon–bone healing is key for ACLR success^34^. This healing process starts with the infiltration of loose, unorganized fibrovascular tissue into the gap between the tendon and bone tunnel, followed by the expression of osteoinductive factors such as bone morphogenetic proteins^35,36^. After 4 weeks, the bone tunnel wall surrounding the tendon graft thickens further^34,35^; then, Sharpey-like fibers, which are the earliest sign of osseointegration into the tendon^37–39^, appear and connect the bone with the tendon. With regard to ACLR in humans, allograft tendons may have inferior remodeling ability in bone tunnels compared with autografts^40^. However, our study has revealed that in case of rats, the BMD of decellularized bovine tendons and autografts of the peritibial tunnels is comparable over time; moreover, the decellularized bovine tendons are speculated to induce autograft-like bone–tendon healing.

Regarding ACLR with autologous tendon grafts, Hays et al. compared the tendon–bone healing process between normal rats and rats injected with liposomal clodronate to deplete the macrophages; the loose fibrous tissue at the tendon–bone interface gradually decreased in width by week 4 in the normal rats, as observed in the present study, but that in the macrophage-depleted rats was significantly narrower at the same time point^35^. Wang et al. observed Sharpey-like fibers at week 8 in a rat ACLR model when using autologous tendon grafts^41^. In our study, Sharpey-like fibers appeared at week 8 in group A, similarly to their results, and in group D at week 4 (i.e., earlier). At the tendon–bone interface, fibroblasts contribute to the formation of Sharpey-like fibers^42^. In our study, Sharpey-like fibers appeared as early as week 4 in group D, similarly to what Hays et al. observed in the macrophage-depleted rat group; they assumed that the depletion of inflammatory mediator-producing macrophages promoted tissue regeneration^35^. Based on our results, the presence of fewer M1 macrophages at an early period in group D compared with group A suggests an advantage of the decellularized tendons for graft–bone healing, like in the macrophage-depleted rats in their study. The earlier formation of Sharpey-like fibers in group D also indicates more rapid cell infiltration and remodeling abilities of group D compared with group A. The Sharpey-like fibers anchor the tendon to the bone, and their development corresponds to the biomechanical strength of the tendon–bone fixation^43^. Although we could not perform biomechanical tests, we may speculate that the decellularized tendons may achieve strong tendon–bone fixation earlier with respect to autografts.

M1 macrophages are activated by type I cytokines such as interferon-γ and tumor necrosis factor-α, and they have antiproliferative, cytotoxic, and proinflammatory activities^44–50^. In contrast, M2 macrophages suppress the production of proinflammatory cytokines and have angiogenic and tissue repair properties^49–52^. During the healing process, these macrophages can plastically change their polarity in response to local stimuli^49,53,54^. Brown et al.^55^ compared four groups of rats that received four types of tissue transplants for abdominal wall defects—cellular autografts, acellular allografts, cellular xenografts, and acellular xenografts. They observed that the acellular tissue transplant group showed a constructive M2-type remodeling response, whereas the cellular tissue transplant group showed an M1-dominant, strong inflammatory response, resulting in connective tissue deposits and scarring, and indicated that the transplantation of tissue containing cellular components, even if they are autologous, leads to an M1-type inflammatory response. Previous studies on tendon injuries reported results similar to those of the current study, with a large increase in the count of M1 macrophages in the first 2 weeks^56,57^, implying that M1 macrophages were involved in acute inflammation^56,58^. In agreement with Brown’s study^55^, we observed M1-dominant inflammation in group A, particularly in the early stages, until the cells (even the autologous ones) in the graft disappeared. Since group D had fewer M1 macrophages than group A in the early periods, it might have also had fewer M1 macrophages in the intra-articular grafts at week 16.

In a rat Achilles tendon repair model, Sugg et al.^56^ reported that the concentration of M2 macrophages became significant at day 28. Similarly, Kawamura et al.^59^ found that M2 macrophages reached maximum accumulation by day 28 in a study on autograft-based bone–tendon healing using the rat ACLR model. In the current study, the number of M2 macrophages in the intra-articular and intratibial tunnel grafts was the highest at week 4 in both the groups. Several studies have reported that an increase in M2 macrophages was associated with the promotion of tendon healing^60,61^. Barboni et al.^60^ reported that the transplantation of human amniotic epithelial cells into injured ovine Achilles tendons increased the expression of M2-related genes and promoted angiogenesis and ECM remodeling. Gelberman et al. found that flexor tendon repair in dogs with adipose-derived mesenchymal stromal cells and recombinant bone morphogenetic protein-12 resulted in increased levels of M2 macrophages, decreased levels of inflammation, and increased levels of proteins involved in ECM production^61^. In this study, significantly more M2 macrophages appeared in the intra-articular grafts of group D at week 4, and numerically more M2 macrophages appeared in the intratibial tunnel grafts at week 8. Throughout the study, the number of M2 macrophages in both the intra-articular and intratibial tunnel grafts of group D was never lower than that of group A. This result indicates that the decellularized tendons induced an M2-dominant host response and earlier cell infiltration into the grafts compared with the autografts. Moreover, the autografts containing cellular components caused M1 type inflammation, and the cell infiltration in group A occurred slower than in group D. These findings imply that decellularized tendons may be a more suitable graft option than autografts.

This study has several limitations. First, the biomechanical properties of explanted decellularized and autologous tendons were not tested because of the considerably small size of the rat knee. Indeed, to obtain reliable tensile test data, it is necessary to develop chucks for the femur and tibia so that the tissue can be placed horizontally with the tensile direction. We are currently conducting ACLR using a bovine-derived decellularized tendon in sheep. Therefore, the biomechanical remodeling properties of the decellularized tendon will be evaluated in future experiments.

Second, it is unclear whether the observation period of 52 weeks in rats is sufficient to thoroughly evaluate in vivo remodeling processes. With regard to ACLR in human autologous hamstring tendons and bone–patellar tendon grafts, a previous study has observed a remodeling process that lasts for 12–24 and 6–12 months, respectively^62^. Fifty-two weeks exceeds the observation period for human bone–patellar tendon grafts but falls within the one for human hamstring tendons. However, remodeling is much slower in human than in animal^63^; therefore, 52 weeks of observation in rats is sufficient to evaluate their in vivo remodeling processes.

This study revealed that bovine tendon-derived decellularized grafts sterilized with ethylene oxide gas after freeze drying have excellent time-series recellularization and tendon–bone integration abilities compared with autologous flexor digitorum longus tendons in the rat ACLR model for up to 52 weeks. The BMD of the peritibial tunnel increased over time and was equivalent to that of the autologous tendons. Furthermore, cell infiltration occurred over time and was equivalent to that occurring in the autologous tendons. Sharpey-like fibers appeared earlier in group D than in group A. Moreover, the number of M2 macrophages, which are responsible for tissue repair, tended to be higher in the decellularized xenograft tendon at 4–8 weeks after ACLR than in the autologous tendon. These findings encouraged us to further study the potential of the decellularized tendon in a cross-species large-animal model.

## Methods

### Ethical compliance

This study was approved by the Animal Research Ethics Committee of Waseda University (approval no.: 2015-A021). All animal experiments complied with the Animal Research: Reporting of In Vivo Experiments guidelines and were carried out following the National Institutes of Health Guide for the Care and Use of Laboratory Animals (NIH publications no. 8023, revised 1978).

### Preparation of decellularized tendon

**Figure 6:**
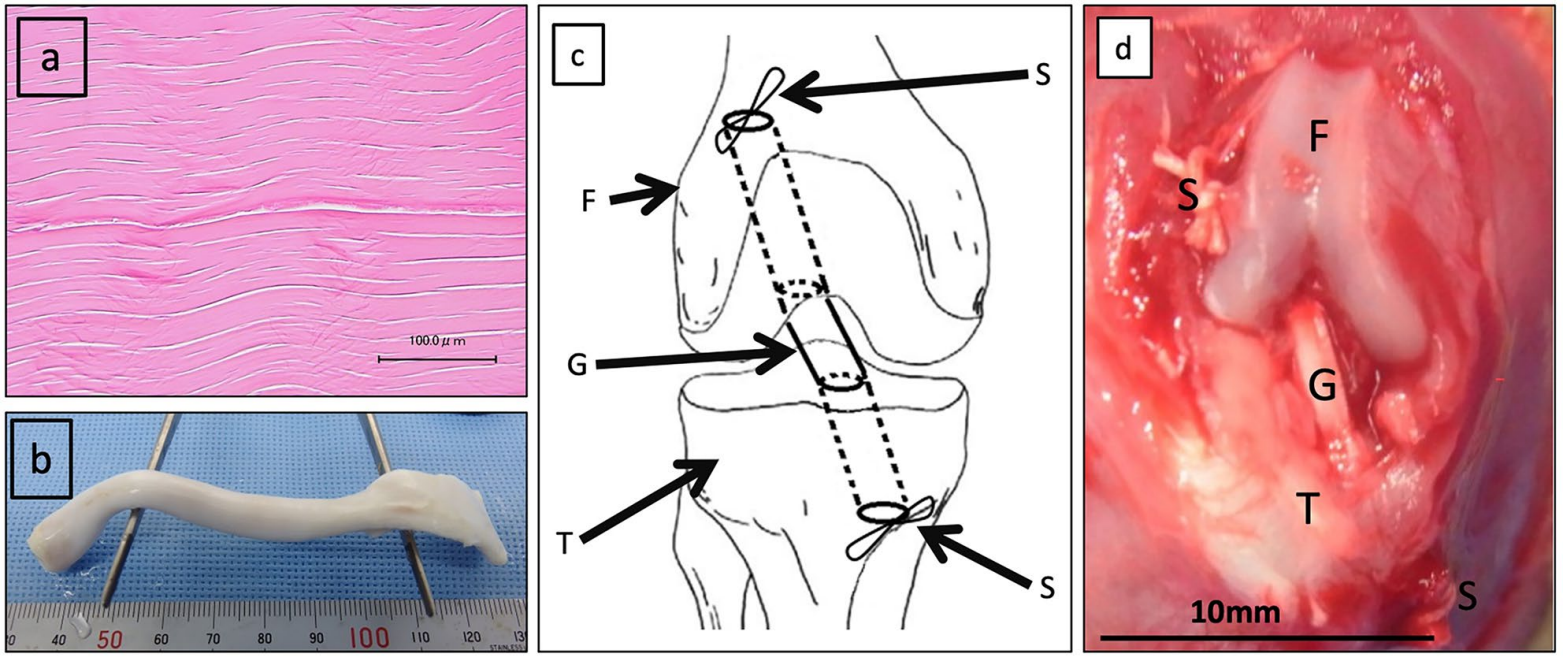
Anterior cruciate ligament reconstruction in rats using decellularized bovine tendon and autologous tendon grafts. (**a**) Hematoxylin–eosin staining of the decellularized bovine tendon. No cell components were observed, and the extracellular matrix and fiber arrangement were preserved. (**b**) The gross appearance of the decellularized bovine tendon after rehydration with physiological saline solution. (**c**) Schematic diagram of the completion of anterior cruciate ligament reconstruction in a rat. (**d**) Gross appearance of anterior cruciate ligament reconstruction in the right knee of the rat at completion. *F* femur, *T* tibia, *G* graft, *S* 3-0 silk thread.

Bovine extensor digitorum tendons were obtained from an abattoir and decellularized using the following procedure^64^. Bovine tendon was incorporated into the pulsatile circulation of physiological saline solution with 1 wt% sodium deoxycholate (Sigma) under a mean flow and pressure of 5 L/min and 80 mmHg, respectively. A microwave was irradiated simultaneously. The temperature of the circulating solution was maintained below 37 °C to prevent thermal denaturation of tissues. The tissues were then treated with Benzonase^®^ nuclease (Merck). The decellularized tissues (Fig. 6a) were freeze-dried for 24 h and then sterilized with ethylene oxide. The tissues were rehydrated with physiological saline solution before use as grafts (Fig. 6b). The residual amount of DNA in the decellularized tendon was assessed using a Quant-iT™ PicoGreen^®^ ds-DNA Assay Kit (Invitrogen). The decellularized tissues had a DNA residue of < 1 ng/mg dry weight, which is significantly less than the value of 50 ng/mg recognized as a safety threshold for decellularized tissue DNA residue^65^. For decellularized tendons sterilized with ethylene oxide gas, the level of residual ethylene oxide and ethylene chlorohydrin in the tendon tissues should be below the limits specified in ISO 10993-7 to ensure safety; for our study, this was confirmed using gas chromatography (Shimadzu Corporation, Kyoto, Japan).

### Experimental animal and evaluation period

Male Sprague–Dawley rats (12 weeks old, 350 ± 50 g) were used. The rats were divided into two groups—group D in which decellularized bovine tendon-derived tissue was used as a graft for right knee ACLR (n = 42) and group A in which the autologous flexor digitorum longus tendon of the right hind limb was used as a graft for ACLR (n = 42). Each group was evaluated at weeks 1, 2, 4, 8, 16, 26, and 52 after ACLR; six animals were evaluated in each period. The BMD of the right peritibial tunnel and the cellularity of the native ACL of the left knee were assessed in six animals with a 0-day implantation period (group N). A total of 90 rats were used for the experiment.

### ACLR in rats

ACLR in rats was performed according to the methods described in previous studies^59,66^. General anesthesia was induced by intraperitoneal injection of 60 mg/kg sodium pentobarbital (Kyoritsu Seiyaku, Tokyo, Japan). If insufficient, additional doses of 6.5 mg each were administered, ensuring that the rats were not overdosed. As a preventative measure, 4 mg/kg cefazolin sodium (LTL pharma, Tokyo, Japan) was injected intraperitoneally. In group D, decellularized bovine tendons were trimmed to obtain a diameter of 1.5 mm and a length of 15 mm, keeping the longitudinal diameter parallel to the fiber direction. In group A, a longitudinal incision was made on the medial side of the ipsilateral lower leg from which the flexor digitorum longus tendon was harvested at a size of approximately 1.5 mm in diameter and 15 mm in length. In each group, a 3-0 silk thread (Akiyama Medical, Tokyo, Japan) was sutured at both ends of the graft. Subsequent procedures were the same for both the groups. The ACL was resected in its natural state. A 1.8-mm diameter Kirschner wire was used to create the femoral tunnel from the footprint of the native ACL to the lateral femoral epicondyle. The tibial tunnel was also created in the same manner. The graft was inserted into the tunnels, and a silk thread was secured to the periosteum around each tunnel aperture, being careful to not loosen the graft (Fig. 6c,d). The joint capsule and skin were sutured using a 5-0 nylon thread (Alfresa Pharma Corporation, Osaka, Japan). Immediately after surgery, the rats were transferred to a warm cage and allowed to move freely upon awakening from anesthesia. None of the rats developed any complications during the observation period.

### Sample harvest

Six rats were euthanized with an overdose of sodium pentobarbital administered via intraperitoneal injection at the time specified above. The meniscus and other ligaments were carefully removed from the femur–graft–tibia complex. The graft integrity was carefully checked using a magnifying loupe. The samples were fixed in 10% formalin solution at 4 °C after rinsing the blood thoroughly using saline.

### Measurement of the BMD of the peritibial tunnel using micro-CT

**Figure 7:**
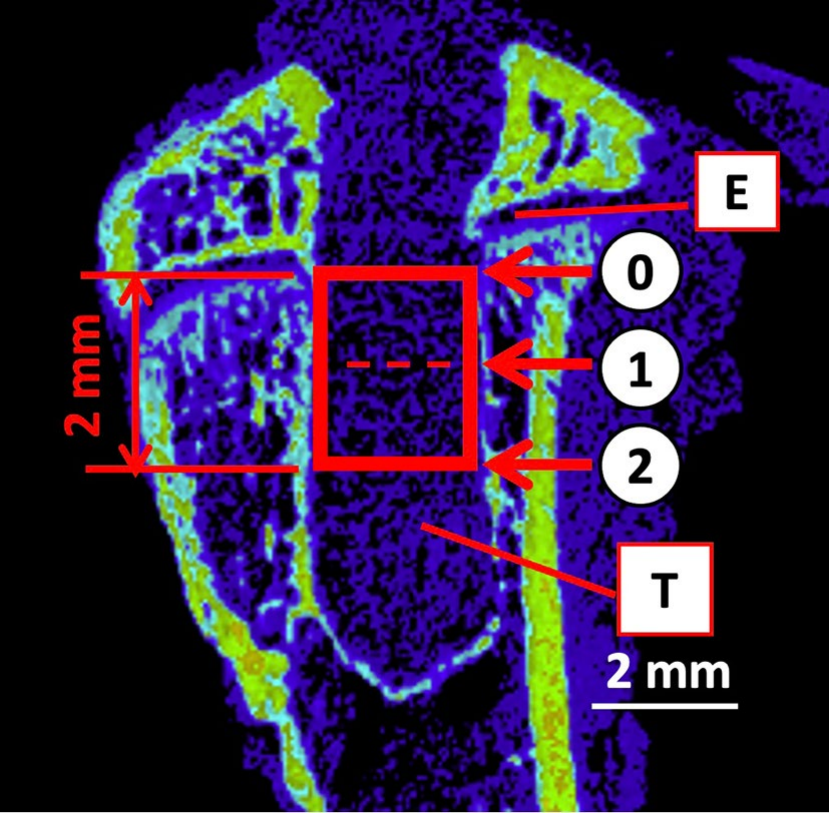
Measurement of bone mineral density at the graft–tibial tunnel interface. The definition of the measurement area of bone mineral density. The CT image shows slices along the long axis of the tibial tunnel. Bone mineral density at the top, middle, and bottom of a region 2 mm distal to the tibial epiphyseal plate was measured. The major and minor tunnel diameters were measured at three cross-sections, namely, the upper (0), middle (1), and lower (2) ends of the region 2 mm distal to the growth cartilage (red square) in the femur–graft–tibia complex. The data on day 0 after anterior cruciate ligament reconstruction were used to determine the baseline diameter of the hole drilled. *CT* computed tomography, *E* epiphysis, *T* tunnel.

Micro-CT images of the femur–graft–tibia complex were taken using a 3D measuring X-ray CT scanner (TDM 1300-IS, Yamato Scientific Co., Ltd, Tokyo, Japan) (set voltage: 45 kV, set current: 236.0 µA, filter: 0.1 mm brass plate, acquisition time: 10 min) to evaluate peritunnel bone reconstruction over time at the interface between the graft and the tibial tunnel. Under the same conditions, phantoms of known density were imaged, and calibration curves were obtained. Based on the calibration curve, 3D BON (RATOC system engineering Co., Ltd, Tokyo, Japan) was used to analyze BMD. In a slice along the long axis of the tibial tunnel, BMD at the top, middle, and bottom of a region 2 mm distal to the tibial epiphyseal plate was measured (Fig. 7).

### Histology

After micro-CT imaging, samples were decalcified using 9% formic acid for 4–6 weeks and then embedded in paraffin. Next, 5-µm-thick longitudinal paraffin sections parallel to the direction of the tibial tunnel and intra-articular graft and parallel to the femoral tunnel were prepared^66^ and stained with hematoxylin–eosin. Fluorescence microscopy (BZ-8100, KEYENCE, Osaka, Japan) was used to observe the whole image (magnification × 2) and local view (magnification × 200). To assess the cellularity of the grafts, the cells inside the intratibial tunnel and intra-articular grafts were counted. The graft–tibial tunnel interface was also analyzed to observe the tendon–bone healing process.

### Immunohistochemical study of macrophage infiltration into the graft

Immunohistochemical staining was performed to investigate the phenotype^55^ of the macrophages (M1, M2) that accumulated in the grafts. Rabbit anti-CCR7 antibody (Cell Applications, San Diego, CA) was used for M1 macrophage staining, and mouse anti-rat CD163 antibody (Serotec) was used for M2 macrophage staining. Staining was performed with reference to a previous study^55^. The macrophages of each phenotype infiltrating the intratibial tunnel and intra-articular grafts were counted.

### Cell count

**Figure 8:**
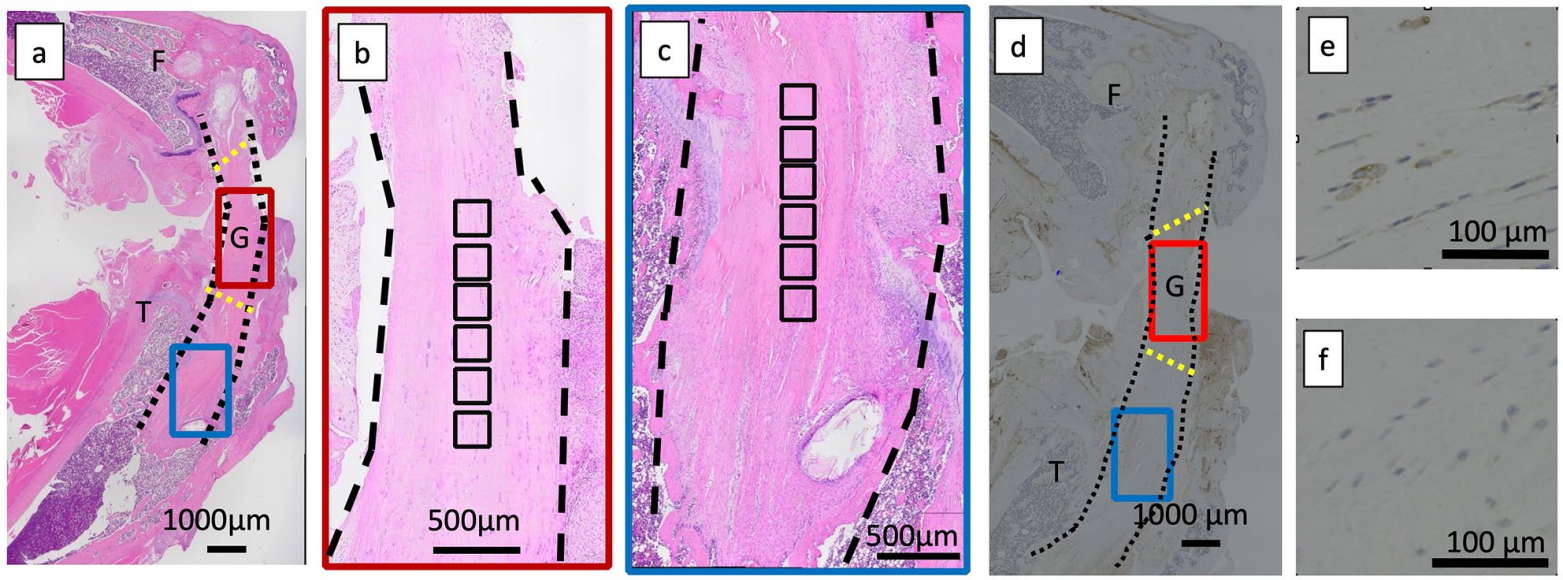
Hematoxylin–eosin staining and immunohistological staining of M1 and M2 macrophages in the femur–graft–tibia complex for quantitative evaluation of cells infiltrated into the decellularized and autologous grafts. (**a**) Overall view of the femur–graft–tibia complex stained with hematoxylin–eosin. (**b**) Magnified view of the intra-articular graft, magnifying the red rectangle in Fig. 7a. (**c**) Magnified view of the graft in the tibial tunnel, magnifying the blue rectangle in Fig. 7a. (**d**) Overall view of the immunohistological staining of M1 and M2 macrophages in the femur–graft–tibia complex. M1 and M2 macrophages in the intra-articular graft (red rectangle) and intratibial tunnel graft (blue rectangle) were counted using the same method used for counting cells observed via hematoxylin–eosin staining. (**e**) M1 macrophages. (**f**) M2 macrophages. The black dashed line shows the surface of the graft. The aperture of the bone tunnel into the joint is indicated by the yellow dashed line. One black square in Fig. 7b,c has a surface area of 200 µm^2^. The cells in six 200-µm^2^ square regions were counted using the ImageJ software (ImageJ, version 1.50i, https://imagej.nih.gov/ij/). *F* femur, *G* graft, *T* tibia.

The entire tissue was observed on a slide using a fluorescence microscope (Fig. 8a). Cells in both the core of the intra-articular graft approximately equidistant from the tibial and femoral tunnel apertures (Fig. 8b) and the core of the intratibial tunnel graft from the tibial tunnel aperture distally (Fig. 8c) were counted. Cell counting was performed using the ImageJ software^67^ in six 200-µm^2^ square regions. Then, the average number of cells in the six squares was used. The number of cells in the native ACL of the left knee was also counted (n = 6) using the same protocol. After immunohistochemical staining, the number of M1 and M2 macrophages was also counted using the same protocol (Fig. 8d–f).

### Statistical analysis

Box-and-whisker plots were used to display the distribution of each data point. The Shapiro–Wilk’s W test was used to test the normality of continuous variables. Depending on whether the distribution was normal, the Student’s t-test or Mann–Whitney U test was used to compare the mean differences between the two groups, and the Kruskal–Wallis test or one-way analysis of variance was used to compare the means of the three groups. When there was a significant difference in the three groups, the Student’s t-test or the Mann–Whitney U test was used to evaluate the difference in the means of each group as a post hoc analysis. Holm’s multiple comparison method was used for p-value correction. Even within the same group, the rats differed if taken at different periods. Therefore, we used the Student’s t-test or the Mann–Whitney U test to evaluate the differences during time within the group. p < 0.05 was considered statistically significant. JMP Pro version 13.2.1 (SAS, Cary, NC, USA) was used for statistical analyses. A post hoc power analysis was performed using the G*Power program, v 3.1.9.3 (Institut für Experimentelle Psychologie, Heinrich Heine Universität, Dusseldorf, Germany). We calculated effect sizes of 1.99 and 2.07 on the differences in, respectively, cellularity and M1 macrophage count between group D and A at week 1. Then, corresponding powers of 0.85 and 0.88 were calculated using these effect sizes, the sample size of six specimens in each group, and the alpha level of 0.05. We found an effect size of 2.41 on the difference in the M2 macrophage count between group D and A at week 4; a corresponding power of 0.95 was similarly obtained.

## Supplementary Information

Supplementary Legends.

Supplementary Video S1.

Supplementary Video S2.

## Data Availability

All data generated or analyzed during this study are included in this published article.
