# Supplementary material for: Time-series biological responses toward decellularized bovine tendon graft and autograft for 52 consecutive weeks after rat anterior cruciate ligament reconstruction
Source: Sci Rep. 2022 Apr 25;12:6751. doi: 10.1038/s41598-022-10713-y (PMC9038763; doi:10.1038/s41598-022-10713-y)
Supplement: Supplementary file 1 — Supplementary Legends. [file 41598_2022_10713_MOESM1_ESM.docx]

**Supplementary material**

Supplementary videos of the bone mineral density of the peritibial bone tunnel at week 4 (Supplementary Video S1) and 52 (Supplementary Video S2) in group D (rats with decellularized bovine tendon graft).
